# Health workers’ perspectives on barriers and facilitators to implementing a new national cervical cancer screening program in Ethiopia

**DOI:** 10.1186/s12905-021-01331-3

**Published:** 2021-05-03

**Authors:** Breanne E. Lott, Atota Halkiyo, Dawit Worku Kassa, Tesfaye Kebede, Abdulkerim Dedefo, John Ehiri, Purnima Madhivanan, Scott Carvajal, Amr Soliman

**Affiliations:** 1 Department of Health Promotion Sciences, Mel and Enid Zuckerman College of Public Health, University of Arizona, 1295 N. Martin Ave., Tucson, AZ 85719 USA; 2 Mary Lou Fulton Teachers College, Arizona State University, 1050 S. Forest Mall, Tempe, AZ 85281 USA; 3 Addis Ababa University, Addis Ababa, Ethiopia; 4 Robe Town Health Office, Bale Robe, Ethiopia; 5 Adama Hospital Medical College, Adama, Ethiopia; 6 Division of Infectious Diseases, College of Medicine, University of Arizona, 1501 N Campbell Ave, Tucson, AZ 85724 USA; 7 Department of Family and Community Medicine, College of Medicine, University of Arizona, 1501 N Campbell Ave, Tucson, AZ 85724 USA; 8 Public Health Research Institute of India, Mysore, India; 9 School of Medicine, The City University of New York, 160 Convent Avenue, New York, NY 10031 USA

**Keywords:** Cancer prevention, Cervical screening, Low-resource setting, Service delivery, Preventive health, Women’s health

## Abstract

**Background:**

Cervical cancer disproportionately affects women in sub-Saharan Africa, compared with other world regions. In Ethiopia, a National Cancer Control Plan published in 2015, outlines an ambitious strategy to reduce the incidence and mortality of cervical cancer. This strategy includes widespread screening using visual inspection with acetic acid (VIA). As the national screening program has rolled out, there has been limited inquiry of provider experiences. This study aims to describe cancer control experts’ perspectives regarding the cancer control strategy and implementation of VIA.

**Methods:**

Semi-structured interviews with 18 participants elicited provider perspectives on cervical cancer prevention and screening. Open-ended interview questions queried barriers and facilitators to implementation of a new national screening program. Responses were analyzed using thematic analysis and mapped to the Integrated Behavioral Model. Participants were health providers and administrators with positionality as cancer control experts including screening program professionals, oncologists, and cancer focal persons at town, zone, and federal health offices at eleven government facilities in the Arsi, Bale, and Shoa zones of the Oromia region, and in the capital Addis Ababa.

**Results:**

The cancer control plan and screening method, VIA, were described by participants as contextually appropriate and responsive to the unique service delivery challenges in Ethiopia. Screening implementation barriers included low community- and provider-awareness of cervical cancer and screening, lack of space and infrastructure to establish the screening center, lack of materials including cryotherapy machines for the “screen-and-treat” approach, and human resource issues such as high-turnover of staff and administration. Participant-generated solutions included additional training for providers, demand creation to increase patient flow through mass media campaigns, decentralization of screening from large regional hospitals to local health centers, improved monitoring and evaluation, and incentivization of screening services to motivate health providers.

**Conclusions:**

As the Ethiopian government refines its Cancer Control Plan and scales up screening service implementation throughout the country, the findings from this study can inform the policies and practices of cervical cancer screening. Provider perspectives of barriers and facilitators to effective cancer control and screening implementation reveal areas for continued improvement such as provider training and coordination and collaboration in the health system.

**Supplementary Information:**

The online version contains supplementary material available at 10.1186/s12905-021-01331-3.

## Background

With one of the highest cervical cancer incidence rates in the world, Ethiopia reported 6,294 new cases of cervical cancer and 4,884 deaths in 2018 [1]. In recent years, the government has pledged support for cervical cancer prevention and provided a framework for scalable screening implementation. Ethiopia’s first Cancer Prevention and Control Plan was published in 2015, outlining a number of ambitious activities to be adopted by the Federal Ministry of Health and Regional Health Bureaus including training various cadres of health providers to provide cervical screening using visual inspection with acetic acid (VIA) and procuring and distributing cryotherapy machines for same-visit treatment of precancerous lesions in VIA + patients as part of a “screen-and-treat” approach free for all women aged 30–49 years old or with other risk factors for cervical cancer [2]. This is the first concerted national effort to establish cervical cancer screening services. A four-year pilot screening program, Addis Tesfa (New Hope), implemented at fourteen sites from 2010 to 2014 laid the groundwork for the Cancer Prevention and Control Plan and demonstrated that VIA is a feasible and appropriate screening method for the Ethiopian context [3]. Then, the former first lady of Ethiopia, Roman Tesfaye Abneh, publicly championed cervical cancer prevention, garnering attention and funding for the cause as well as establishing a National Cancer Control Taskforce. Since the national screening program was started in 2015, more than 250 health facilities across Ethiopia have started screening and the Ministry plans to increase that number to 800 in the next phase of scale-up (Personal communication, Ministry of Health, August 9, 2019).

Still, uptake of cervical cancer remains low. Facility- and community-based surveys have found screening utilization ranging from 0 to 24.8% in populations across the country [4–23]. Barriers to uptake of screening such as low community awareness, lack of experience with screening, accessibility of screening services, long distances to health facilities and transportation issues, fear or embarrassment associated with cervical exams, other negative attitudes about screening, and misconceptions about cervical cancer have been documented, primarily through questionnaires distributed to women [4, 11, 24–27]. Despite the challenges, women indicate a high willingness to be screened; 86.2% of HIV-positive women in an Addis Ababa facility-based study were willing to be screened if free of cost and yet only 17% reported that they had ever received a provider recommendation for cervical cancer screening [11]. Providers anecdotally report many challenges to implementing screening, particularly if screening has never been or is currently not offered at their health facility. Aside from the Addis Tesfa implementation report and another quality improvement project, there are no studies to the best of our knowledge documenting provider perspectives on screening program implementation in Ethiopia to date [3, 28]. The aim of this study, therefore, was to describe provider perspectives on cervical cancer prevention and control efforts in Ethiopia, with a focus on cervical cancer screening using visual inspection with acetic acid. Key informants, including screening program managers and cancer prevention focal persons, were interviewed to explore barriers and facilitators to screening implementation.

## Methods

Eighteen semi-structured key informant interviews were conducted to elicit health workers’ perspectives on cervical screening as a cancer prevention strategy in one geographic region of Ethiopia from May to July 2019.

### Study setting

**Fig. 1:**
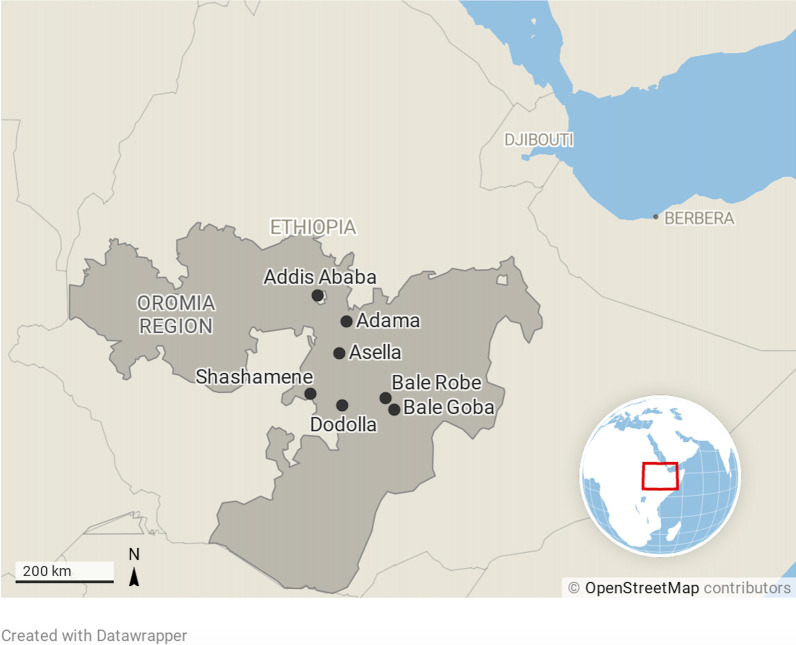
Map of study sites

The study was conducted in several administrative zones of the Oromia region of Ethiopia: Shewa, West Arsi, Arsi, and Bale. Oromia is the largest administrative region of Ethiopia by land mass and by population, accounting for about 35% of the country’s total population [29]. Study sites are located in Eastern Oromia, 90 to 500 km to the south and east of the capital city, Addis Ababa, along major transportation corridors as seen in Fig. 1. The locations were purposively selected due to presence of cervical cancer experts (see participant and recruitment below) and feasibility of data collection (site accessibility with public transportation) while also attempting to include a variety of perspectives from locations with well-established vs. new screening programs. Additionally, key personnel were recruited in Addis Ababa, where the country’s only Oncology Center and the Federal Ministry of Health are located, to represent a national-level perspective.

### Participants and recruitment

Key informants with specialized knowledge of cervical cancer screening, prevention, and treatment were selected. Participants were any cadre of health worker or health administrators that could be described as cancer experts given their positionality directly offering cervical cancer screening or treatment or overseeing the provision of cervical cancer services. This included cervical screening program managers, other workers offering cervical cancer screening for at least two years, gynecologic and clinical oncologists, Medical Directors, Hospital Chief Executive Directors, and town, zonal, or national cervical cancer focal persons. The participants could have been employed in primary, secondary, or tertiary government health facilities or health offices. Other inclusion criteria were that the participant must be aged 18 years or older, could have any level of formal education or any degree, and could identify as any race, ethnicity, gender, or religion.

Informants were recruited face-to-face in their places of employment. Since few individuals in Ethiopia meet the inclusion criteria, purposive snowball sampling was used to identify key individuals with the appropriate qualifications and then solicit the help of study participants to identify additional participants [30]. First, the in-country research team (BEL, ABH, DWK, AD, TK) made site visits to health facilities known to offer cervical cancer screening. No list of health facilities offering screening is publicly available and incomplete and inaccurate reporting led to significant discrepancies between communities that reported presence of screening services and those actively performing screening at the time of the study. Through consultation with local experts (ABH, DWK, AD, TK), a community-based hear-say approach was deemed most accurate for screening community identification. Meetings with hospital administrators and the screening program were used to identify specific health workers at each facility that may be eligible to participate as well as additional facilities or participants at other facilities that may be contacted. Vertical recruiting occurred, where we worked our way up and down the administrative chain of cervical cancer screening oversight in a particular location with strong social ties between participants as well as horizontal recruiting where weaker social ties or hear-say were used to direct us to other screening sites [31]. A recruitment script was used to share information about the study’s purpose, expected benefits and risks, and participant eligibility. No individuals were excluded or refused to participate. Recruitment continued until data saturation was reached, as evidenced by informational redundancy in latter interviews [32]. The researchers reflexively discussed key themes at the conclusion of each interview and the decision to cease recruitment was subjectively made when themes became increasingly similar between participants.

### Interviews

Semi-structured interviews were conducted in-person at participants’ places of employment or other private, off-site locations at the convenience of the participant. An interview guide with pre-determined interview questions was used (Additional file 1: Appendix 1). The interview questions were created in consultation with a gynecologic oncologist (DW) and a screening program manager for content validity. Probing questions were also used to elicit more detailed information from informants, at the discretion of the researchers, and evolved throughout the study based on emergent themes from previous interviews [33]. Interviews were always conducted by at least two co-authors (BEL and ABH) who both have previous training in qualitative research methods. At some research sites, additional co-authors also participated in interviews (AD, TK). Interviews were conducted in three languages, according to participant preference: Amharic (official, national language), Afan Oromo (official, regional language), and English. Participants and interviewers may have used multiple languages in the same interview, as preferred to fully communicate meaning. This multi-lingual approach to the data collection, which involved use of a multi-cultural research team rather than use of interpreters and language translation enhances the integrity of the qualitative data [34]. All interviews were audio-recorded, with participant permission. Interviews ranged from 20 to 60 min. Participants were compensated for their time, with a prepaid mobile phone calling card at the conclusion of the interview valued at 100 Ethiopian birr (~ $3 USD).

### Analysis

Thematic analysis was conducted to identify key emergent themes from the interviews [35]. Two researchers (BEL and ABH) jointly listened to the audio-recordings for familiarization and used open-coding to create meaning around excerpts. Then the codes were categorized into emergent themes using discussion and consensus (BEL and ABH). Data were analyzed in the language in which the interviews were conducted. Supporting quotes were transcribed in English using meaning-based translation; the combination of oral and written transcription is used to systematically validate key concepts [34, 36]. The Integrated Behavioral Model (IBM) was used as a framework during analysis to understand how specific barriers and facilitators could ultimately impact a provider’s intent to offer screening or actual ability to implement cervical cancer screening [37]. Emergent themes were mapped to the IBM constructs and are presented in a table. Findings are presented using the key emergent themes, supporting quotes, and are presented in text and tables as appropriate. This study is reported in accordance with Consolidated Criteria for Reporting Qualitative Research (COREQ) [38].

### Ethical considerations

This study was approved by the University of Arizona Institutional Review Board and the Oromia Regional Health Bureau.

## Results

Participants were mostly male (67%) and were working at cervical cancer screening programs as screeners or managers (50%), were cancer focal persons at town, zonal, or federal levels (22%), treated cancer as oncologists (17%), or were in decision-making positions as hospital administrators (11%). The majority of participants worked at general hospitals (39%) or teaching and referral hospitals (44%). Participant characteristics are reported in Table 1.

**Table 1:** Interview participant characteristics

Characteristic	Number of participants	Percent (%) of total participants (n = 18)
Location		
Addis Ababa	3	16.7
Adama	2	11.1
Shashamene	5	27.8
Dodolla	1	5.6
Bale Goba	2	11.1
Bale Robe	2	11.1
Asella	3	16.7
Workplace/facility type		
General hospital	7	38.9
Referral/teaching hospital	8	44.4
Town health office	1	5.6
Zonal health office	1	5.6
Federal Ministry of Health	1	5.6
Title/position		
Screening program professional	9	50.0
Cancer focal person	4	22.2
Oncologist	3	16.7
Hospital administrator	2	11.11
Screening program length (n = 12)		
No functional screening program	4	33.3
Less than 1 year	1	8.3
1–3 years	4	33.3
3 + years	3	25.0
Sex		
Male	12	66.7
Female	6	33.3

### Attitudes about cervical cancer and appropriateness of screening methods

All screening programs were using visual inspection with acetic acid (VIA), while some had the availability of a cryotherapy machine to provide treatment on site and others had to refer to another facility for cryotherapy. Participants felt that VIA was an appropriate screening method for the context in which they were providing screening services, citing reasons of low-cost, low-tech, quick and easy interpretation, and ability of all cadres of health professionals to offer VIA screening with some training. They expressed hope to be able to address cervical cancer in Ethiopia through cervical screening. Motivation to prioritize this health issue stemmed from undeniably high cervical cancer incidence and mortality, which are receiving more attention recently, as well as the fact that most cervical cancer is preventable. Many had witnessed the poor state of women affected by advanced stage cervical cancer and held deeply personal convictions to prevent other women from experiencing the same thing.

Cervical cancer can be preventable. If it is screened and if the vaccine is given on time, it is a preventable disease. But if people are not screened and don’t receive cryotherapy, it is such a drastic disease, you know. – Participant 13.

If we focus on prevention, we reduce the burden on Black Lion (Oncology Center). Two, we might keep so many mothers from dying and we reduce their painful suffering. – Participant 15.

VIA is so good for low-income countries. We are serving it freely so cost-wise, it is good. Also, it takes little time for screening and treatment even with cryotherapy. They don’t have to come back. It is simple, it is cheap. – Participant 4.

Visual inspection is the way forward, at least for now. I think it’s impossible to implement nationwide any other method like pap smear or HPV testing but at large health facilities it is possible. – Participant 1.

### Cervical cancer and screening awareness

Participants repeatedly cited the newness of cervical cancer as a health topic in their country, both as a motivating factor driving innovation and action as well as a barrier since the awareness of the issue is low among the community and even health professionals. In fact, the greatest barrier, most cited by participants, was lack of awareness of cervical cancer. Within the community, an overall sense of ignorance about cervical cancer was compounded by perceived fear of cancer as a deadly disease with no hope of prevention or recovery and therefore no chance of medical intervention. Low utilization of preventive health service was described, with health providers explaining that typically patients only go to the hospital when feeling ill and when the illness is extreme, not for routine care such as various screenings. Participants felt like mass media campaigns, such as those previously conducted for other health issues like HIV prevention, could be an effective strategy for increasing community awareness. Among providers, participants expressed a desire for integration of cervical cancer training into all formal education in addition to scale up of short-term, intensive workshops specifically about cervical cancer prevention and screening with VIA.

The majority of the patients don’t understand what the disease is, the course of the disease, or the outcomes. – Participant 3

The majority of our people are coming from the rural countryside, they don’t know about cervical cancer. Even the health professionals, they don’t know. – Participant 9

This thing is very new. Even professionals do not know about cervical cancer. Therefore, information should be disseminated in gatherings like ‘idir’ or mourning meetings. Like March 8^th^ (International Women’s Day). I beg and participate to share information. – Participant 6.

The one who comes or who we invite for screening normally is the healthy woman. In our culture, healthy women leaving her work/chores, leaving everything and coming to be seen, it is not a custom. Therefore, since there is such problem, it is by high pressure or pushing that they come to our office. A person can come only if they are sick. – Participant 16.

### Space and materials to offer screening

Other frequently cited barriers to screening implementation included environmental challenges such as lack of a proper screening space or materials. Some facilities reported lack of any screening space while others reported concerns about the inadequacy of their screening space due to cleanliness or privacy. At one facility, the lack of space had prevented the trained personnel from offering any cervical cancer screening in the two years since they had received the training. Repurposing of buildings (one hospital had been opened in an old hotel building and one referral hospital had previously been a general hospital) was cited as a limitation since the very infrastructure was created for a different purpose than its current use. Participants stressed that because of the intimate nature of the cervical examination and the types of questions asked during counseling, both cleanliness and privacy should be enhanced for a screening room when compared with other types of service delivery. Successful screening programs often had a detached room, behind or adjacent to the main hospital building, perhaps within proximity to the antiretroviral therapy clinic which both ensured greater patient privacy and allowed for convenient integration and synergy between the two services. One participant even described making his service mobile, taking his screening supplies to other departments in the hospital to ensure maximum convenience and comfort for the patients.

The patients want to screen but the patients need confidentiality. For confidentiality, I go there. And here, when I screen, I close the door. Women do not want to show their genital area so I am systematic about it. The patient history asks about multiple sexual partners. In our culture, it is very shameful. So women say ‘please don’t tell any other person.’ – Participant 6.

We see the cervix of the woman, how can we check that in the room where dust comes in from every direction?…You check the human cervix – a cervix can be infected by little things. And there are secrets. – Participant 10.

Providers reported a long delay between VIA training and receipt of a cryotherapy machine which is required to effectively implement the “screen-and-treat” approach. It was not uncommon to wait for two or three years to receive the material. Participants described this as problematic for a number of reasons including high staff turnover, deterioration of knowledge and skills to provide the training, and decreased motivation to launch the screening program in the interim. Some had not received it to date and in some cases cited this as the reason why they were unable to offer the screening at all. The Ministry of Health recently procured enough machines to theoretically provide them to 1500 facilities, but supply-chain problems and lack of coordination between federal, regional, and local administrations were reported. Participant-provided solutions for this challenge included only training individuals for whom an entire screening program supply was available, including a cryotherapy machine immediately available to the health facility, as well as refresher training for providers who did not have the opportunity to immediately practice VIA screening upon completion of their initial training. Another issue related to the cryotherapy machines was maintenance. Accessibility and availability of the tips used tor cryotherapy treatment were a concern. Lastly, other materials such as the acetic acid and speculums were also in short supply. Participants reported paying for acetic acid out-of-pocket and obtaining speculums from other departments.

We are waiting for that equipment to screen. He [regional distributor] told me… if it reaches us, we will distribute it immediately but it never reached to us from the head office (in Addis Ababa). – Participant 7.

Let me tell you, when you are trained in a particular thing, you have to implement it immediately. Unless you implement immediately, you’ll start forgetting it. It has been three years, see! There’s no materials, no materials. They made us wait for three years. We said we don’t even want to work because we wanted them to give us re-training. During that time, we were trained very well. It is difficult to conduct now after three years. – Participant 10.

### Human resources and administrative support

A major determining factor of environmental challenges was support from hospital administration. There was considerable variation in the budgets and reported support for cervical cancer screening programs at each facility visited. Some administrators had assigned providers to work full-time or dedicated shifts at the screening clinic while other providers did so in their free time or at their own will. In such cases, the providers reported that choosing to staff the screening center actually reduced their opportunity for working overnight duty shifts for which they are paid extra, thereby putting VIA screening shifts in direct opposition with their ability to earn income. This alongside a limited number of staff that were trained to offer the service in the first place resulted in screening rooms that sometimes were unstaffed and closed. Admittedly, providers recognize that when patients attempt to seek screening and find a closed room, they will probably never return again for screening. Several participants described high turnover of Medical Directors as a challenge, since one Director may support the program but then the next does not. Similarly, administrators described high turnover of staff as a challenge to continuity of the screening programs. Participants wished to see some form of incentivization for providers to staff the screening center, which requires administrative prioritization of the issue and may alleviate some of the staffing problems.

There’s no specific incentive for that person. They’re going to be leaving that post, they move somewhere else. The money that they are supposed to get working during night shift, they are not going to get that at the screening clinic. – Participant 1.

After taking training when you go to follow up, they will be gone. The room is there, the material is there, but the skilled one (trained health provider) is gone. – Participant 12.

### Policy, monitoring and evaluation

Just as providers viewed administrative support within their facilities as fickle, they also described support from the highest level at the Ministry of Health as “unpredictable” in nature. Providers recognized the current support for cervical cancer screening, and non-communicable diseases in general, but were also wary of the extent to which the actual support would reflect the support pledged by documents such as the National Cancer Control Plan and whether the support expected next year could be anticipated based on the support received in the current year. Participants described ways in which programs would suddenly change or cease to exist; the government used to subsidize chemotherapies and then suddenly stopped and breast cancer diagnostic tests used to be available in-country and then ceased to be available. In terms of the Integrated Behavioral Model, this challenge can be understood as negatively impacting the salience of the behavior over time.

The participants received the national policy positively while expressing a desire for greater monitoring and evaluation as well as accountability. They also wanted to be consulted by the policy-makers and said that they were in the process of establishing professional organizations to give greater voice to the experts and to use their positionality to inform evidence-based policies and practices.

Those things are available at the good will of the Ministry of Health…I think the strategy that they have on paper, it looks really nice. The only problem is the implementation. It’s taking time maybe? They’re not trying to enforce it in the right way? – Participant 3.

Follow up is also needed. For example, in the first round I conducted the job (screening). Where should I report? To the region? To the zone? After I called the federal office to ask, she appointed a focal person for each zone. I consider that even as an achievement and feel happy about it…There is no follow-up at the country level. There is negligence for cervical cancer. For example, if the federal ministry of health gives it attention like HIV, but this has no emphasis yet. – Participant 6.

### Sustaining screening programs

Of the 12 participants that were screening program professionals, hospital administrators, or otherwise affiliated with a health facility that was reported to have a screening program, there was a difference in interview themes based on how long the screening program had been in operation. Most of the participants (n = 5) worked at facilities with screening centers that had been functioning for two months to three years. Two facilities had professionals that were trained to offer VIA services but were unable to establish screening services due to previously mentioned challenges. On the other end of the spectrum, there was one screening site in particular which opened several years before the others, serving as a pilot program even before the 2015 National Cancer Control Plan was published. Three participants from this site described challenges to sustaining their service over time, in contrast to the other sites that described challenges getting their screening programs up and running. They mentioned that they used to screen a lot of women and that their service delivery had diminished significantly over time. Staff and leadership turnover was one challenge that disrupted continuity and quality of services. They also described a desire for what they coined “demand creation” where communication and media campaigns increase awareness and interest in the community. Past media campaigns were described as successful, especially when they educated men and women about cervical cancer; the interviewees recalled many women who came in for screening at the prompting of their husbands.

You hate your work when you have nothing to report because there is no patient flow. You feel embarrassed inside. You get satisfaction if there are patients and you work, even if there's no other incentive. – Participant 15.

These participants also had enough experience referring cervical cancer patients for treatment to speak about challenges in the continuum of care. With only one cancer treatment center in the capital city, access issues are prevalent with long wait times from referral to treatment for women who even reach treatment center at all. This demotivated the providers from screening more women because they were not confident in the system’s ability to treat any cancer cases detected. Lack of communication after referral meant they were unsure what their patients’ outcomes were.

Out of ten women screened, one has cancer. There is a high prevalence if a lot of women are screened. But there is a weak referral process. They are working to create a better internal referring structure or linkage between departments in the hospital. – Participant 16.

They did describe collaboration with some other organizations that provide financial support for referrals such as covering transportation and lodging costs to get to the treatment center in Addis Ababa, which was a best practice since it removed environmental barriers to treatment access. This facility was also in the process of establishing their own treatment center, which they were hopeful about.

### Other barriers and facilitators to screening

Table 2 presents additional themes from the interviews, organizing provider perceived barriers and facilitators to implementation of cervical cancer screening according to constructs from the Integrated Behavioral Model. In terms of provider self-efficacy to overcome challenges, intrinsic motivation and good rapport with patients and other health providers were described as important. Health Extension Workers were acknowledged as a positive partner in the community, who should be educating and referring women for screening at health facilities. Gender and religion were cited as patient identities that should be considered when providing responsive health counselling. Providers saw an opportunity to involve male partners and peers to influence individual decision-making as well as societal norms about screening. Another major theme was inconsistency in the cervical cancer and VIA training received by each provider, with some cancer focal persons receiving one lecture within a two-day orientation on non-communicable diseases while providers received training that ranged from one to three weeks in length. Participants called for a standardized curriculum that included skill-based modules and a practical attachment so that they could practice VIA screening under supervision. They also wished to have some sort of certification upon completion of training.

**Table 2:** Barriers and facilitators to cervical cancer screening implementation, using the Integrated Behavioral Model (IBM)

IBM construct	Barriers	Facilitators and strategies to overcome barriers
Attitudes	Patient attitudes: “Why go to the hospital if I am healthy?” contributes to low patient flow/demand of services Patient attitudes: Low awareness and misconceptions Gender and religion were cited as factors that shape patient attitudes about screening Provider attitudes: “Why screen if we don’t have the cryotherapy machine?” prevents facilities from offering screening until the material arrives Provider attitude: “What happens if she has cervical cancer? We are afraid to screen because then we have to treat.” Lack of treatment options, long time from diagnosis to treatment, and poor prognosis are demotivating factors	Mass media awareness raising campaigns can promote preventive care seeking Providers feel that they are able to overcome misconceptions with proper counselling Have female health workers available to screen and consider gender and religion when counselling Strong referral networks can be used to screen at one facility and conduct cryotherapy at another Provider experience: Seeing women suffer from advanced cervical cancer was a strong motivating factor Provider perspective: Knowing that cervical cancer is preventable was a strong motivating factor to offer screening
Experiential (feelings about behavior)		
Instrumental (behavioral beliefs)		
Norms	Lack of monitoring and evaluation (M&E) made providers feel like there was low accountability to actually screen after receiving training Providers expressed that cervical screening was a service they offered in their “extra time” without additional incentive, not their primary job function The success of a screening clinic is highly dependent on the motivation of one or a few individuals Sometimes male partners were engaged in a woman’s medical decision-making, concern over male partner approval was especially relevant for cryotherapy as sex is prohibited for some time after	Government support for the issue at the national level created positive expectations to offer screening which could be enhanced by M&E Administration that prioritizes the service can offer incentives and assign workers to the screening clinic to ensure adequate coverage Providers with high motivation to offer screening should be selected to receive the training Male partners can be strategically engaged to improve screening norms Screened women can offer peer support and education for other women
Injunctive (others’ expectations)		
Descriptive (others’ behaviors)		
Personal agency	Environmental constraints such as lack of materials and space were major limiting factors Perspective: Things happen at the “good will of the Ministry of Health” Policy can be influenced by personal and political motives, not evidence-based Poor coordination between facilities and local, regional, and federal government can hinder progress Other job duties make it difficult to prioritize cervical screening, providers too busy with acute care duties	Some providers overcame environmental constraints by borrowing materials from other units or facilities or paid for supplies out-of-pocket Consistent funding, training, oversight, and policy are needed for steady growth of services Providers wanted to be consulted by the Ministry and have a greater voice in policy-making Creation of professional organizations, task forces, and committees can create space for more diverse voices, representation, and collective action Building strong patient rapport allowed providers to overcome low community awareness
Perceived control		
Self-efficacy		
Knowledge and skills to perform screening	Some cadres of health workers may be more or less equipped to offer cervical screening Training varied greatly from a two-day orientation to a three-week intensive skill-based training with a practical attachment at a hospital Confidence to perform screening decreased over long periods of time between training and initiation of screening services	Midwives were viewed as especially proficient, emergency surgeons were not preferred Training should be standardized Refresher training should be made available:
Salience of behavior and Habit	Changing political landscapes can disrupt progress Declining patient flow over time was a demotivating factor	When the former First Lady championed the cause, attention was garnered for cervical cancer with some lasting effects Steady patient flow, through collaboration with Health Extension Workers and community-based education, motivated screening clinics to continue providing the service
Environmental constraints	*Human resources* High turn-over of providers and Medical Directors Screening centers are understaffed *Space and infrastructure* Inadequate space or no room available to screen Inadequate privacy or cleanliness in available space *Materials* No cryotherapy machine received, distribution issues Tips for the cryotherapy machine were limited in size and easily damaged (not easily replaced) Other materials (speculum, acetic acid, examination table) were also difficult to procure	*Human resources* Training more than 1–2 providers in each facility to offer screening means that screening can continue if a single provider leaves or is off-duty *Space and infrastructure* Providers screen at night to maximize privacy Providers offer mobile screening service, taking materials to another space in the hospital *Materials* Having spare parts, such as tips, available could facilitate maintenance over time Providers procured materials (i.e. speculum) from other units or facilities Providers purchased their own acetic acid Some facilities were able to redirect funds to purchase some supplies such as gloves *Other* VIA was viewed as an appropriate screening method because it considered environmental constraints

## Discussion

In this study, health care providers in Ethiopia described barriers and facilitators to implementation of a new cervical cancer screening program. A major perceived barrier was lack of awareness at the community-level and among general health providers. Low awareness is a well-documented barrier, though previous research has shown that educational interventions for women are not effective for increasing uptake of cervical cancer screening in sub-Saharan Africa [39]. Understanding provider perspectives on barriers, particularly those other than low awareness, can be used to develop alternative interventions to initiate screening, scale up screening, and increase screening uptake in the community. Other common barriers to trained providers initiating screening were environmental constraints such as a lack of space and insufficient materials. A previous study of the cervical cancer screening pilot program found that 4 out of 14 pilot facilities said their screening room was too small, though none had raised issues of cleanliness of the space, and many facilities reported a shortage of supplies such as speculum or forceps [3]. Wavering dedication of both the providers and administration of the hospital and the federal Ministry of Health were issues. Disruptions in funding, attention, lack of follow-up, and inconsistency in training and messaging from health administrations limited the perceived control and self-efficacy of providers in this study. They cited support from administration as a major facilitator while also describing how changes in administration led to uncertainty about whether their efforts would be appreciated and prioritized by the next generation of leadership. Similarly, Shiferaw et al. characterized high staff turn-over as a threat, with 26% of workers trained to offer VIA (n = 20/77) no longer working at the screening site at the follow-up [3]. Many of these same challenges have been reported elsewhere in sub-Saharan Africa [40–44].

Throughout this study, it was clear that providers had a plethora of experience to share including strategies for overcoming difficulties and best practices for implementation of cervical cancer screening. Yet, they had little opportunity to share experiences with each other or to effectively report their experiences. Participants thought that creation and strengthening of professional associations would be one possible solution to come together around this topic. They described several roles that associations could play in furthering cancer prevention work in the country including developing content and curriculum, providing professional development trainings, creating a support network for professionals in all regions, engaging with a variety of stakeholders including patients and survivors, liaising with the Ministry of Health for greater participation in policy-making, and fundraising. Existing associations addressing some of these elements included the Family Guidance Association of Ethiopia and the Midwifery Association. Other partners that were also acknowledged for their significant contributions included Pathfinder and Grounds for Health.

One question that was raised by several participants was “are hospitals the best place for cervical cancer screening?” As a preventive health service, VIA was sometimes deprioritized in hospital settings. Administrators and providers at one health facility where a midwife had been trained to offer screening but had not initiated the service in the two years following the training described the constraints of their facility, including inadequate patient waiting areas since the building was a repurposed hotel, as well as the lack of staffing as major challenges. They exceed other health facilities in number of deliveries each year and report frequently having women laboring in the grass. They also explained that they sometimes have trauma patients laying on the ground outside and that if any room was to become available, it would make more sense for them to dedicate the space to these pressing issues instead of offering preventive screening services. They expressed desire to offer screening but felt that perhaps another type of health facility like a health center would be better suited to offer such services, as they aren’t dealing with the huge influx of emergency cases. The Ministry of Health does have plans to cascade cervical cancer screening to smaller health facilities, like health centers. It appears that the initial rollout was most feasible in the largest cities, at established hospitals, and that the next phase of the plan will decentralize screening, reaching more remote locations and smaller facilities as the practice is further dispersed. At least one participant in this study indicated a desire to become a trainer, through training of trainer (TOT), so that he could assume more responsibility for increasing the number of trained providers in his zone. This is just one way that the government can leverage the already trained individuals during scale-up and decentralization process and has been successfully done in other settings [45]. This could ultimately make screening more accessible to women, as they would have less distance to travel to reach the health facility and could also potentially increase the quality of services rendered. Further research is needed to compare patient outcomes and patient and provider preferences with regard to facility type.

Another topic raised in the interviews was perceived appropriateness of VIA for cervical cancer screening in the Ethiopian context. All participants characterized VIA as appropriate citing reasons of easy implementation that was appropriate for low-resource settings and could be performed by practically any cadre of trained health provider. When prompted further to discuss any perceived negative aspects of VIA compared to other types of screening, providers elaborated that there may be room to expand use of human papillomavirus (HPV) testing in the future but that at the moment only VIA was scalable. Pap smears are available at private clinics in some urban centers in Ethiopia, but they are expensive and difficult to analyze given the shortage of pathologists available to interpret the sample results. As the world moves toward a 70% screening coverage goal, declared in the WHO Strategy of Cervical Cancer Elimination, HPV testing can be used for primary screening of cervical cancer and its precursors, allow health providers to focus their screening efforts on high-risk HPV-positive women, and improve screen-and-treat outcomes [46, 47]. HPV testing has been successfully implemented in many low-resource settings, alone and in conjunction with additional screening methods [48, 49] and implementation models used in other settings such as the use of onsite analysis which can minimize loss to follow up should be further explored for use in Ethiopia.

One study of facility-based, self-administered HPV testing in Butajira, Ethiopia reported 84.1% screening uptake among intervention participants compared with 50.5% uptake among participants who received the same community-based educational intervention and were referred for facility-based VIA [50]. Another study used focus group discussions with women in rural Ethiopian communities to describe acceptability of self-sampling and found that women felt they would be able to perform the task of collecting a sample in their home or other private location and liked the self-sampling as it decreased their fear and embarrassment of screening [51]. These studies suggest that self-sampling and community-based sampling could be important advances to further explore to increase screening uptake in Ethiopia. Since the COVID-19 pandemic, when many non-essential health services have become even more inaccessible, the importance of community-based screening strategies has only been further illuminated. Much work remains for scaling up and strengthening VIA service delivery as well as introducing HPV testing into the country’s screening strategy.

As more facilities offer cervical cancer screening, increased attention should be paid to developing referral pathways and coordinating service delivery across sites. In this study, we found that many of the facilities would report a particular challenge that could be solved by collaborating with the next closest facility. In two sites that are just a 20-min bus ride apart, one site, with a large staff and substantial patient pool, reported that they were unable to offer screening because they had no cryotherapy machine while the other, which had a cryotherapy machine, described staffing challenges and low patient demand. Each facility was attempting to solve their own challenges in silos when it was apparent to outside observers that partnership between the two facilities could give each facility an increased capacity to solve their issue and to ultimately provide a coordinated service to the shared community.

The findings from this study can be used by various stakeholders, including individual providers and health institutions, as well as the Federal Ministry of Health to inform cervical cancer screening policies and practices in Ethiopia. This is the first study, to our knowledge, to provide an in-depth description of providers’ perspectives on the implementation of a new national cervical cancer screening program. At the time of this study, VIA was the only screening method recommended by the national screening program and was widely accepted by the participants. As other screening methods become cheaper and more readily accessible, the lessons learned from implementation of VIA can be carried over to implement screening with other methods like HPV testing as well [52]. The findings reported herein are timely as they bring the voices of multiple cancer prevention stakeholders to the forefront as Ethiopia moves beyond the initial Cancer Control Plan’s implementation phase and prior to a second iteration being published or made widely available. A National Cancer Control Taskforce is currently reviewing the plan with the Ministry; one of this study’s authors (DW) serves on the Taskforce.

### Limitations

Given the small pool of cervical cancer experts in Ethiopia who meet the inclusion criteria and the data collection method of semi-structured interviews, this study is susceptible to self-report or social desirability bias whereby participants favorably present their knowledge and experiences [53]. Participant anonymity was protected to the extent possible, by including information about it in the informed consent process and by only reporting de-identified data. It was also made clear to the subject that their participation was voluntary and that findings would in no way be used as evaluation of their performance. This anonymity encouraged participants to speak freely during the interviews without fear of repercussion for any comments that could be construed as negative. As evidenced by the findings, many challenges were openly discussed. Member checking, where participants are given an opportunity to review findings and provide feedback, was not performed since participant names and contact information was not recorded and there was, therefore, no way to follow up with the interviewees at a later time when data analysis was complete. While participants did not have an opportunity to respond, the bi-national research team ensured that Ethiopian perspectives were represented in study design and planning, data collection, and interpretation as described below.

Another potential limitation is the discordance between the first author’s identity (nationality, race, gender, etc.) and those of the participants which could influence interactions during data collection and interpretation during data analysis. To minimize this limitation and to maximize the cultural responsiveness of this study, a multi-cultural research team was coordinated with at least one Ethiopian-born investigator involved in each stage of the study. All interviews were conducted by at least two researchers, one of whom was fully trilingual (ABH). Data analysis was also conducted in a pair (BEL and ABH), with consensus required for identification of key qualitative themes to allow for culturally nuanced interpretation of the findings.

## Conclusions

Cancer control experts, including screening program managers and cervical cancer focal persons, shared their views on a new national cervical cancer prevention strategy and implementation of cervical cancer screening with visual inspection with acetic acid in Ethiopia. Challenges described included low community awareness of cervical cancer, low provider awareness of cervical cancer, lack of space and equipment to offer screening, and variable support from administration at institutional, local, regional, and national levels. Providers were able to overcome many challenges and envisioned their role in cancer prevention as both important and impactful, reducing the pain, suffering, and mortality of women in their communities.

## Supplementary Information

**Additional file 1:** Appendix 1_Interview Questions.pdf. Interview Questions. Prepared questions used to guide semi-structured interviews with participants.

**Additional file 2:** Lott_COREQ Checklist.pdf. COREQ Checklist. Completed COREQ checklist for manuscript submitted 27 March 2021.

## Data Availability

The qualitative data generated and analysed during the current study are not publicly available to protect the anonymity of our participants but deidentified data may be made available from the corresponding author on reasonable request.

## Abbreviations

HPV: Human papillomavirus
IBM: Integrated behavioral model
TOT: Training of trainers
VIA: Visual inspection with acetic acid

## References

[1] World Health Organization. International Agency for Research on Cancer and World Health Organization: GLOBOCAN 2018: estimated cervical cancer incidence and mortality. 2018.

[2] Ethiopian Federal Ministry of Health. National Cancer Control Plan 2016–2020. 2015.

[3] Shiferaw N, Salvador-Davila G, Kassahun K, Brooks MI, Weldegebreal T, Tilahun Y (2016). The single-visit approach as a cervical cancer prevention strategy among women with HIV in Ethiopia: successes and lessons learned. Glob Health Sci Pract.

[4] Tilahun T, Tulu T, Dechasa W (2019). Knowledge, attitude and practice of cervical cancer screening and associated factors amongst female students at Wollega University, western Ethiopia. BMC Res Notes.

[5] Bante SA, Getie SA, Getu AA, Mulatu K, Fenta SL (2019). Uptake of pre-cervical cancer screening and associated factors among reproductive age women in Debre Markos town, Northwest Ethiopia, 2017. BMC Public Health.

[6] Assefa AA, Astawesegn FH, Eshetu B (2019). Cervical cancer screening service utilization and associated factors among HIV positive women attending adult ART clinic in public health facilities, Hawassa town, Ethiopia: a cross-sectional study. BMC Health Serv Res.

[7] Nigussie T, Admassu B, Nigussie A (2019). Cervical cancer screening service utilization and associated factors among age-eligible women in Jimma town using health belief model, South West Ethiopia. BMC Womens Health.

[8] Erku DA, Netere AK, Mersha AG, Abebe SA, Mekuria AB, Belachew SA (2017). Comprehensive knowledge and uptake of cervical cancer screening is low among women living with HIV/AIDS in Northwest Ethiopia. Gynecol Oncol Res Pract.

[9] Habtu Y, Yohannes S, Laelago T (2018). Health seeking behavior and its determinants for cervical cancer among women of childbearing age in Hossana Town, Hadiya zone, Southern Ethiopia: community based cross sectional study. BMC Cancer.

[10] Tefera F, Mitiku I (2017). Uptake of cervical cancer screening and associated factors among 15–49-Year-old women in Dessie Town. Northeast Ethiopia J Cancer Educ.

[11] Bayu H, Berhe Y, Mulat A, Alemu A (2016). Cervical cancer screening service uptake and associated factors among age eligible women in Mekelle zone, Northern Ethiopia, 2015: a community based study using Health Belief Model. PLoS ONE.

[12] Aweke YH, Ayanto SY, Ersado TL (2017). Knowledge, attitude and practice for cervical cancer prevention and control among women of childbearing age in Hossana Town, Hadiya zone, Southern Ethiopia: community-based cross-sectional study. PLoS ONE.

[13] Kasa AS, Tesfaye TD, Temesgen WA (2018). Knowledge, attitude and practice towards cervical cancer among women in Finote Selam city administration, West Gojjam Zone, Amhara Region, North West Ethiopia, 2017. Afr Health Sci.

[14] Belete N, Tsige Y, Mellie H (2015). Willingness and acceptability of cervical cancer screening among women living with HIV/AIDS in Addis Ababa, Ethiopia: a cross sectional study. Gynecol Oncol Res Pract.

[15] Ruddies F, Gizaw M, Teka B, Thies S, Wienke A, Kaufmann AM (2020). Cervical cancer screening in rural Ethiopia: a cross- sectional knowledge, attitude and practice study. BMC Cancer.

[16] Dessalegn MB (2020). Cervical cancer screening uptake and associated factors among HIV-positive women in Ethiopia: a systematic review and meta-analysis. Adv Prev Med.

[17] Kasim J, Kalu A, Kamara B, Alema HB (2020). Cervical cancer screening service utilization and associated factors among women in the Shabadino District. Southern Ethiopia J Cancer Epidemiol.

[18] Tekle T, Wolka E, Nega B, Kumma WP, Koyira MM (2020). Knowledge, attitude and practice towards cervical cancer screening among women and associated factors in hospitals of Wolaita Zone. Southern Ethiopia Cancer Manag Res.

[19] Nega AD, Woldetsadik MA, Gelagay AA (2018). Low uptake of cervical cancer screening among HIV positive women in Gondar University referral hospital, Northwest Ethiopia: cross-sectional study design. BMC Womens Health.

[20] Aynalem BY, Anteneh KT, Enyew MM (2020). Utilization of cervical cancer screening and associated factors among women in Debremarkos town, Amhara region, Northwest Ethiopia: Community based cross-sectional study. PLoS ONE.

[21] Gebregziabher D, Berhanie E, Birhanu T, Tesfamariam K (2019). Correlates of cervical cancer screening uptake among female under graduate students of Aksum University, College of Health Sciences, Tigray, Ethiopia. BMC Res Notes.

[22] Woldetsadik AB, Amhare AF, Bitew ST, Pei L, Lei J, Han J (2020). Socio-demographic characteristics and associated factors influencing cervical cancer screening among women attending in St. Paul's Teaching and Referral Hospital, Ethiopia. BMC Womens Health.

[23] Fentie AM, Tadesse TB, Gebretekle GB (2020). Factors affecting cervical cancer screening uptake, visual inspection with acetic acid positivity and its predictors among women attending cervical cancer screening service in Addis Ababa, Ethiopia. BMC Womens Health.

[24] Getachew S, Getachew E, Gizaw M, Ayele W, Addissie A, Kantelhardt EJ (2019). Cervical cancer screening knowledge and barriers among women in Addis Ababa, Ethiopia. PLoS ONE.

[25] Chaka B, Sayed AR, Goeieman B, Rayne S (2018). A survey of knowledge and attitudes relating to cervical and breast cancer among women in Ethiopia. BMC Public Health.

[26] Shiferaw S, Addissie A, Gizaw M, Hirpa S, Ayele W, Getachew S, Kantelhardt EJ, Assefa M, Jemal A (2018). Knowledge about cervical cancer and barriers toward cervical cancer screening among HIV-positive women attending public health centers in Addis Ababa city. Ethiopia Cancer Med.

[27] Dulla D, Daka D, Wakgari N (2017). Knowledge about cervical cancer screening and its practice among female health care workers in southern Ethiopia: a cross-sectional study. Int J Womens Health.

[28] Shiferaw N, Brooks MI, Salvador-Davila G, Lonsako S, Kassahun K, Ansel J, Osakwe C, Weldegebreal T, Ahmed I, Asnake M, Blumenthal PD (2016). Knowledge and awareness of cervical cancer among HIV-infected women in Ethiopia. Obstet Gynecol Int.

[29] Price JT, Asgary R (2016). Implementation and feasibility of an adapted two-stage visual inspection with acetic acid/cryotherapy-based cervical cancer screening programme for HIV-infected women in Addis Ababa. Ethiopia Eur J Cancer Care (Engl).

[30] The World Factbook 2020: Ethiopia. Washington, DC: Central Intelligence Agency, 2020. Accessed 31 October 2020. https://www.cia.gov/library/publications/the-world-factbook/geos/et.html.

[31] Noy C (2008). Sampling knowledge: the hermeneutics of snowball sampling in qualitative research. Int J Soc.

[32] Geddes A, Parker C, Scott S (2018). When the snowball fails to roll and the use of ‘horizontal’networking in qualitative social research. Int J Soc Res Methodol.

[33] Saunders B, Sim J, Kingstone T, Baker S, Waterfield J, Bartlam B (2018). Saturation in qualitative research: exploring its conceptualization and operationalization. Qual Quant.

[34] Longhurst R (2003). Semi-structured interviews and focus groups. Key methods in geography.

[35] Inhetveen K. Translation challenges: Qualitative interviewing in a multi-lingual field. Qualitative Sociology Review. 2012;8(2).

[36] Braun V, Clarke V (2006). Using thematic analysis in psychology. Qual Res Psychol.

[37] Larson ML (1984). Meaning-based translation: A guide to cross-language equivalence.

[38] Montaño DE, Kasprzyk D (2015). Theory of reasoned action, theory of planned behavior, and the integrated behavioral model. Health behavior: Theory, research and practice.

[39] Tong A, Sainsbury P, Craig J (2007). Consolidated criteria for reporting qualitative research (COREQ): a 32-item checklist for interviews and focus groups. Int J Qual Health Care.

[40] Lott BE, Trejo MJ, Baum C, McClelland DJ, Adsul P, Madhivanan P, Carvajal S, Ernst K, Ehiri J (2020). Interventions to increase uptake of cervical screening in sub-Saharan Africa: a scoping review using the integrated behavioral model. BMC Public Health.

[41] Munthali AC, Ngwira BM, Taulo F (2015). Exploring barriers to the delivery of cervical cancer screening and early treatment services in Malawi: some views from service providers. Patient Prefer Adherence.

[42] Black E, Hyslop F, Richmond R (2019). Barriers and facilitators to uptake of cervical cancer screening among women in Uganda: a systematic review. BMC Womens Health.

[43] McFarland DM, Gueldner SM, Mogobe KD (2016). Integrated review of barriers to cervical cancer screening in sub-Saharan Africa. J Nurs Scholarsh.

[44] Rosser JI, Hamisi S, Njoroge B, Huchko MJ (2015). Barriers to cervical cancer screening in rural Kenya: perspectives from a provider survey. J Community Health.

[45] Rahman R, Clark MD, Collins Z, Traore F, Dioukhane EM, Thiam H, Ndiaye Y, De Jesus EL, Danfakha N, Peters KE, Komarek T (2019). Cervical cancer screening decentralized policy adaptation: an African rural-context-specific systematic literature review. Glob Health Action.

[46] McCree R, Giattas MR, Sahasrabuddhe VV, Jolly PE, Martin MY, Usdan SL, Kohler C, Lisovicz N (2015). Expanding cervical cancer screening and treatment in Tanzania: stakeholders’ perceptions of structural influences on scale-up. Oncologist.

[47] Global strategy to accelerate the elimination of cervical cancer as a public health problem. Geneva: World Health Organization. 2020.

[48] Denny L, Kuhn L, Hu CC, Tsai WY, Wright TC (2010). Human papillomavirus-based cervical cancer prevention: long-term results of a randomized screening trial. J Natl Cancer Inst.

[49] Sankaranarayanan R, Nene BM, Shastri SS, Jayant K, Muwonge R, Budukh AM (2009). HPV screening for cervical cancer in rural India. N Engl J Med.

[50] Woo YL (2019). The feasibility and acceptability of self-sampling and HPV testing using Cepheid Xpert® HPV in a busy primary care facility. J Virus Erad.

[51] Gizaw M, Teka B, Ruddies F, Abebe T, Kaufmann AM, Worku A, Wienke A, Jemal A, Addissie A, Kantelhardt EJ (2019). Uptake of cervical cancer screening in Ethiopia by self-sampling HPV DNA compared to visual inspection with acetic acid: a cluster randomized trial. Cancer Prev Res.

[52] Brandt T, Wubneh SB, Handebo S, Debalkie G, Ayanaw Y, Alemu K, Jede F, von Knebel DM, Bussmann H (2019). Genital self-sampling for HPV-based cervical cancer screening: a qualitative study of preferences and barriers in rural Ethiopia. BMC Public Health.

[53] Sankaranarayanan R (2014). Screening for cancer in low-and middle-income countries. Ann Glob Health.

[54] Van de Mortel TF (2008). Faking it: social desirability response bias in self-report research. Aust. J. Adv. Nurs..

