# Supplementary material for: Health workers’ perspectives on barriers and facilitators to implementing a new national cervical cancer screening program in Ethiopia
Source: BMC Womens Health. 2021 May 3;21:185. doi: 10.1186/s12905-021-01331-3 (PMC8090515; doi:10.1186/s12905-021-01331-3)
Supplement: Supplementary file 1 — Additional file 1: Appendix 1_Interview Questions.pdf. Interview Questions. Prepared questions used to guide semi-structured interviews with participants. [file 12905_2021_1331_MOESM1_ESM.docx]

**Appendix 1. Interview questions**

1. Tell me about your job.
   - What is your title?
   - How many years have you been practicing medicine/in the health field?
   - What type of facility do you work at?
   - Do you perform cervical cancer screenings?
2. I want to learn about cervical cancer in Ethiopia, what can you tell me?
   - Is cervical cancer a major problem in Ethiopia, why do you think so?
   - How are cancer in general and cervical cancer perceived by the public?
   - How are they perceived by health professionals?
3. The Ethiopian Ministry of Health published a cancer control plan for years 2016-2020. It seems like there is a concerted effort to address cancer in Ethiopia. What has your experience been? Are you aware of the plan and what movement have you seen around this topic in recent years?
   - Tell me about new programs, interventions, or training opportunities you have observed in recent years.
   - How do you feel about the government’s efforts?
4. How did you learn about the topic of cervical cancer?
   - How do you think your experience is similar or different from other Ethiopian health care providers?
5. Do you think that cervical cancer screening is important in Ethiopia and why or why not?
6. What types of screening are available/common here?
   - What do you think are the pros and cons of each method?
7. What are some challenges to providing cervical cancer screening in the Ethiopian context?
   - What are the challenges for providers, for patients, and for the health system?
   - Most women in Ethiopia have never had a cervical exam before, why do you think that is?
8. What things make it easier for you (or others) to provide cervical cancer screening (facilitators)?
   - Are there best practices from your experience that you could share with other health providers in Ethiopia?
   - Are there best practices from Ethiopia that you think other countries could learn from?
9. When you think about the next five years of cancer control in Ethiopia, from 2020-2025, what recommendations would you make for the country?
   - What needs to be done to get more providers to offer screening?
   - Do you think the government should continue training providers to perform visual inspection with acetic acid (VIA)? Why? Are other methods feasible?
10. Do you have anything else you want to tell me about cervical cancer or cervical cancer screening?
